# CEIRR Risk Assessment Pipeline executive reports on H5N1 highly pathogenic avian influenza 2.3.4.4b, swine H1 1B.2, and H9N2 low pathogenicity avian influenza B4.7.2

**DOI:** 10.1128/jvi.00545-26

**Published:** 2026-06-03

**Authors:** The Centers of Excellence for Influenza Research and Response Risk Assessment Pipeline, Tavis Anderson

**Affiliations:** St Jude Children's Research Hospital, Memphis, Tennessee, USA

**Keywords:** pandemic, outbreak, spillover, zoonotic, risk assessment, influenza

## Abstract

The Centers of Excellence for Influenza Research and Response (CEIRR) Risk Assessment Pipeline (RAP) integrates surveillance, phenotypic analysis, and computational modeling across six CEIRR centers to evaluate the pandemic potential of influenza A viruses. By generating coordinated data sets from wild and domestic animals and linking them to viral evolution and functional traits, CEIRR RAP supports the Centers for Disease Control and Prevention’s and the World Health Organization’s risk-assessment efforts. The RAP’s data packages thereby enable evidence-based prioritization of global influenza preparedness and response strategies.

## COMMENTARY

## WHAT IS THE CEIRR RISK ASSESSMENT PIPELINE?

The Centers of Excellence for Influenza Research and Response (CEIRR) is a network of academic scientists funded by the National Institute of Allergy and Infectious Diseases at the National Institutes of Health. A core mission of the CEIRR program is to advance outbreak preparedness and response through research. This mission is pursued through the integrated activities of dozens of investigators, which together form the CEIRR Risk Assessment Pipeline (CEIRR RAP). Drawing on expertise from each of the six CEIRR centers, the CEIRR RAP includes virologists, immunologists, computational biologists, clinicians, veterinarians, epidemiologists, and ecologists. These investigators operate within four interconnected sub-groups (Wild Animal Surveillance, Domestic Animal Surveillance, Phylodynamics and Computational Modeling, and Phenotypic Characterization) that together ensure robust data collection across diverse topic areas important for assessing the threats posed by influenza viruses.

## WHY IS THE CEIRR RAP NEEDED?

As we have seen four times since 1918, influenza A viruses circulating in non-human hosts can cause pandemics. These viruses are genetically diverse and circulate in a multitude of avian and mammalian species, producing widely varying ecological dynamics. In theory, any influenza A virus to which humans lack widespread immunity could adapt to spread in humans and cause a pandemic. In practice, there are ecological and evolutionary barriers that limit the likelihood of these events. The function of the CEIRR RAP is to produce and analyze data that allow the complexity of potential pandemic threats to be navigated based on evidence.

## WHAT HAVE CEIRR INVESTIGATORS FOUND?

The CEIRR RAP has undertaken since 2024 to produce data packages on influenza A virus lineages with suspected outbreak potential. These packages comprise published or unpublished data generated by CEIRR investigators that are relevant to assessing the risk posed by the lineage in question.

To date, the CEIRR RAP has produced four data packages: one on H5N1 highly pathogenic avian influenza of the 2.3.4.4b sub-clade, one on the D1.1 genotype of this same sub-clade, one on swine H1 1B.2 lineage viruses, and one on H9N2 low pathogenicity avian influenza of the B4.7.2 lineage. To facilitate their use by a broad audience, we have synthesized the information in these data packages to produce an executive summary of each. These reports are not intended to provide a comprehensive review of the literature on each viral lineage but rather summarize the data generated by CEIRR investigators. Both the full data packages and the corresponding executive summaries are accessible to the public on the CEIRR website at https://www.ceirr-network.org/resources/rap.

To contextualize the information, the group has also incorporated phenotypic data on specific H5N1 strains into a Nextstrain build. Nextstrain is a publicly accessible resource that curates viral genetic data into interactive phylogenetic trees that enable visualization viral evolutionary patterns. The CEIRR RAP Nextstrain page, https://www.ceirr-network.org/resources/nextstrain, includes a filter to highlight strains that have undergone *in vitro* or *in vivo* characterization. Hovering over the specific strain provides details on phenotypic observations and reveals a link to published data or indicates the CEIRR center that generated unpublished data. This tool not only allows users to consider the risks posed by newly evolved stains but also allows researchers to identify phylogenetic sub-groupings that are under-characterized.

## HOW DOES THE CEIRR RAP INTERACT WITH RELATED ACTIVITIES AT CDC AND WHO?

Both the U.S. Centers for Disease Control and Prevention (CDC) and the World Health Organization (WHO) run a formalized process to assess the pandemic risk posed by specific influenza A viruses. At CDC, the Influenza Risk Assessment Tool (IRAT) (1) is used, and, at WHO, the related Tool for Influenza Pandemic Risk Assessment (TIPRA) (2) is employed. These tools rely on knowledge of the virus–including its phenotypes, diversity, geographic distribution, and host range—and knowledge of immunity to the virus in human populations. Based on this information, IRAT and TIPRA risk assessment exercises yield quantitative estimates of the likelihood of a pandemic and its expected impact. The CEIRR RAP data packages are specifically designed to complement the work of IRAT and TIPRA by providing pertinent, high-quality data that can be employed in their risk assessment exercises.

## OUTLOOK

As new threats arise and long-standing threats evolve, the CEIRR RAP will continue to advance public health readiness through research. Using the mechanisms outlined above, the outputs of these efforts will continue to be shared directly with public health partners and broadly with all interested parties. In these ways, the CEIRR RAP will advance robust and evidence-based influenza risk assessment.
